# Beyond Conventional Transcriptional Regulation Function: FOXP3 as an Integrative Hub for Chromatin Interactions and Protein Complexes in Immune Regulation

**DOI:** 10.3390/biology15030254

**Published:** 2026-01-30

**Authors:** He Chang, Yongqiang Wang, Li Gao, Shijun J. Zheng

**Affiliations:** 1 State Key Laboratory of Veterinary Public Health and Security, Beijing 100193, China; bs20223050471@cau.edu.cn (H.C.);; 2 Key Laboratory of Animal Epidemiology of the Ministry of Agriculture, Beijing 100193, China; 3 College of Veterinary Medicine, China Agricultural University, Beijing 100193, China

**Keywords:** FOXP3, regulatory T cells, transcription factor, DNA binding

## Abstract

As the lineage-defining transcription factor for regulatory T cells (Tregs), FOXP3 plays a critical role in maintaining immune homeostasis. However, FOXP3 has not been found to regulate the expression of immune suppressive cytokines so far, and the specific molecular mechanisms of its function remain an ongoing debate. Emerging evidence reveals that FOXP3 has functions beyond its traditional role as a DNA-binding transcriptional regulator. It possesses unique characteristics distinct from other Forkhead (FKH) family members or lineage-defining transcription factors, including its distinctive sequence recognition preferences, multimeric structure, and function as a central hub for multiprotein complex assembly. Critically, FOXP3 mediates long-range chromatin interactions through its DNA-bridging capacity and multimerization. Furthermore, it integrates environmental signals by interacting with diverse context-dependent cofactors to dynamically regulate gene expression. This review focuses on recent advances elucidating these novel functions of FOXP3, aiming to provide a reference for a deeper understanding of its multifaceted roles in Treg biology.

## Simple Summary

Regulatory T cells (Tregs), a distinct subset of CD4^+^ T lymphocytes, play indispensable roles in restraining immune responses to sustain immune tolerance and prevent excessive inflammation. The Forkhead box transcription factor FOXP3 serves as the master regulator for the development and function of regulatory T cells. Understanding how FOXP3 exerts its regulatory functions across various biological settings remains a major research focus. Current findings reveal that FOXP3 interacts with diverse context-dependent cofactors and utilizes its DNA-bridging capacity to mediate long-range chromatin interactions. Thus, FOXP3 represents a novel class of transcription factor that functions as a multimodal interaction hub, integrating diverse signals to dynamically coordinate global gene expression, a paradigm distinct from its previously defined role as a conventional and single-acting transcription factor.

## 1. Introduction

Regulatory T cells (Tregs) represent a specialized CD4^+^ T lymphocyte subpopulation essential for maintaining immune tolerance and suppressing autoimmune pathologies [1,2,3]. CD25^+^ T cells were originally identified as key regulators of autoimmunity in 1995, and depleting these cells induced multi-organ inflammation in mice, indicating that CD4^+^CD25^+^ Tregs were essential for immune suppression, thereby preventing autoimmune diseases [4].

The identification of *Foxp3/FOXP3* mutations as the cause of the scurfy mouse phenotype and the human IPEX (Immune dysregulation, polyendocrinopathy, enteropathy, X-linked) syndrome in 2001 established FOXP3 as an essential regulator of immune homeostasis. (In this review, we follow the standard nomenclature where FOXP3/*FOXP3* refers to the human protein/gene and Foxp3/*Foxp3* to the murine orthologs, respectively. In general, FOXP3 is used to refer to the transcription factor generally across species unless otherwise specified [2,5,6,7]). Then, in 2003, FOXP3 was identified as the lineage-defining transcription factor that determines Treg cell identity [8,9,10]. Loss of FOXP3 protein in differentiated Tregs causes their functional impairment (initially elucidated in murine models) [11]. Genetic deficiency of *Foxp3* in scurfy mice induces fatal lymphoproliferation, while ectopic expression confers suppressive function to conventional CD4^+^ T cells (Tconv) [8,9,10]. Collectively, these findings established FOXP3 as the indispensable molecule for Treg development and function [12,13,14]. The 2025 Nobel Prize in Physiology or Medicine was awarded to the researchers for their discoveries establishing the field of peripheral immune tolerance [15]. The identification of Tregs and FOXP3 revealed the cellular and molecular basis for preventing autoimmunity and fundamentally advanced our comprehension of immune homeostasis.

FOXP3^+^ Tregs employ diverse mechanisms to suppress immune responses [16,17]. They express co-inhibitory molecules, including CTLA-4 and LAG-3, modulating antigen-presenting cell (APC) activity [18,19]. They also secrete inhibitory cytokines such as IL-10, TGF-β, and IL-35, which broadly suppress target cells [20,21,22]. Furthermore, their constitutive high expression of CD25 (also known as IL-2Rα, the high-affinity chain of the IL-2 receptor (IL-2R)) facilitates the sequestration of IL-2 from the local microenvironment, thereby suppressing the survival and proliferation of target cells [16,23,24,25].

The process of T cell differentiation is tightly regulated, critically involving epigenetic modifications at key genomic sites that govern lineage determination [26,27]. Naïve CD4^+^ T cells differentiate into distinct mature T helper (Th) subsets (Th1, Th2, Th17, Treg) under specific cytokine-mediated signals that induce lineage-defining master transcription factors [28]. IFN-γ and IL-12 drive TBX21 (also known as T-bet) expression for Th1 polarization. IL-4 induces GATA3 for the Th2 lineage. TGF-β combined with IL-6 triggers RORγt to generate Th17 cells, while TGF-β induces FOXP3, directing the commitment to the Treg phenotype [26,27,28,29]. This lineage specification is enforced by strict epigenetic control. Precise epigenetic control over the *FOXP3* locus, mediated by both DNA methylation and histone modifications, establishes a distinct transcriptional network. This network drives stable *FOXP3* expression and consequently sustains the regulatory phenotype characteristic of Tregs [30,31].

Despite more than two decades since the identification of FOXP3 as the master transcriptional regulator for Tregs, there are still many controversies regarding its precise molecular functions and mechanisms of action. Indeed, it is these questions that drive the research in this field with rapid progress. Comparative analyses of gene expression profiles between FOXP3^+^ and FOXP3^−^ T cells indicate that a large number of Treg-specific transcriptomes are FOXP3-independent [12,13,32,33,34,35,36,37]. FOXP3 is neither required nor adequate to determine Treg identity, fundamentally challenging the traditional point of view [30,37].

A persistent debate revolves around whether FOXP3 acts predominantly as a transcriptional activator [33,38,39,40], repressor [41,42,43,44], or exhibits both functions depending on partner proteins [45], or indirectly via modulating the expression of other transcription factors [46]. Furthermore, does FOXP3 have definitive target genes? What is its specific DNA-binding sequence? Or does it mainly serve as a cofactor, responding to varied environmental cues and interacting with distinct transcription factors to dynamically regulate Treg function? What factors ultimately dictate the functionality of FOXP3? These questions have emerged as major active research priorities in recent years. This review systematically summarizes recent functional studies on FOXP3 as the key Treg transcriptional regulator, providing a reference for future research in this field.

## 2. FOXP3 Evolution: Conserved Core, Adaptive Structure in Treg Function

The genetic architecture of *FOXP3* displays significant evolutionary conservation across vertebrates, while adapting to species-specific immunological requirements. In humans and mice, *FOXP3/Foxp3* localizes to the X chromosome and encodes a Forkhead (FKH)-domain protein essential for maintaining immune tolerance and orchestrating tissue homeostasis [47]. Comprehensive phylogenetic analyses confirm the presence of *FOXP3* orthologs in mammals, fish, and birds, highlighting its ancient evolutionary role in maintaining immune tolerance [48,49,50,51]. In case of poultry, the identification of functional FOXP3 in chickens (*Gallus gallus*) reveals conserved protein domains critical for Treg-mediated immunosuppression, despite approximately 310 million years of evolutionary divergence from mammals [52].

*FOXP3* is regulated by multiple *cis*-regulatory elements (CREs) located within its promoter and enhancer regions (specifically, conserved non-coding sequences (CNS) CNS0, CNS1, CNS2, and CNS3) (initially elucidated in murine models) [53,54,55,56]. These regulatory elements harbor binding sites for transcription factors whose activation is triggered by extracellular signaling pathways, including those from TCR (T cell receptor), CD28, TGF-β receptor (TGF-βR), and IL-2R engagement [30,57,58,59]. Stable *FOXP3* expression in Treg cells depends on demethylation of its Treg-specific demethylated region (TSDR) within the CNS2 region of the *FOXP3* locus [2,54,60,61]. CNS2 deletion abrogates *FOXP3* expression during Treg expansion and impairs Treg stability [62,63,64,65]. CNS1 controls *FOXP3* expression induced peripherally but not in the thymus, while CNS3 potently increases the probability of *FOXP3* gene expression during both thymic and peripheral differentiation of Tregs [54]. CNS0, bound by the genome organizer SATB1, acts as a super-enhancer for *FOXP3* induction in double-positive thymocytes during Treg development [56,66,67,68]. Environmental cues, such as dietary metabolites, play a critical role in regulating *FOXP3* expression. A prime example is the regulation by short-chain fatty acids (SCFAs), particularly butyrate. Mechanistically, SCFAs act as histone deacetylase (HDAC) inhibitors, promoting histone acetylation at the *Foxp3* locus and enhancer regions (e.g., CNS1). This epigenetic modification increases chromatin accessibility, thereby facilitating stable *Foxp3* expression (initially elucidated in murine models) [69,70,71]. A recent study confirmed that SCFAs similarly augment the differentiation and function of human Tregs [72]. FOXP3 shows distinct species-specific expression patterns between mice and humans [73]. In mice, it is expressed exclusively and stably in Tregs [8,10]. In comparison, humans have both constitutive FOXP3 expression in Tregs and transient FOXP3 expression in Tconv cells upon stimulation with CD3/CD28 plus IL-2 [74,75,76]. The core mechanism underlying these differences involves the negative non-coding sequence (NS-) [73]. Murine NS- contains high-affinity binding motifs for repressive transcription factors (e.g., Egr2, Tfdp1), which stringently block Foxp3 induction in Tconv cells. Conversely, human NS- exerts weaker repression, and human Tconv FOXP3 induction relies on positive CREs (CNS0, Tconv cell-specific positive non-coding sequence (NS+)) and shared transcription factors (GATA3, STAT5) that maintain chromatin accessibility at these CREs. CNS1-3 are dispensable for FOXP3 induction in human Tconv cells [73]. The divergent strength of NS-repression and species-specific CRE-transcription factor circuitry results in the distinct FOXP3 expression dynamics observed across mice and humans [73].

FOXP3 belongs to the Forkhead box (FOX) family of transcription factors, specifically the FOXP subfamily. In mammals, this subfamily comprises four members: FOXP1, FOXP2, FOXP3, and FOXP4 [77]. FOXP3, conserved among mammals, shares functional domains between humans and mice with 86.5% amino acid sequence homology. These domains include an N-terminal proline-rich region (ProR), a zinc finger (ZF), a leucine zipper (LZ), and an FKH domain [78]. The gene comprises one non-coding exon (−1) and 11 coding exons (1–11) [79]. Despite this conservation, notably, more than 50% of FOXP3-bound DNA regions are human-specific [78,79,80,81,82]. Recent studies have highlighted distinct regulatory mechanisms in human Tregs. For instance, the transcription factor MEOX1, directly regulated by FOXP3, serves as a critical regulator of human Treg activity [83]. Whereas rodents exclusively express the full-length isoform [78,84], humans produce multiple alternatively spliced isoforms [85,86,87]. The predominant isoforms are full-length FOXP3 (FOXP3-FL) and exon 2-deficient variant (FOXP3-ΔE2); minor isoforms lack exon 7 (FOXP3-ΔE7) or both exons 2 and 7 (FOXP3-ΔE2ΔE7) [79,88]. Among these isoforms, FOXP3-ΔE2 lacks the motif required for interaction with Th17-associated transcription factors RORα and RORγt [89,90,91]. Although FOXP3-ΔE2 is capable of generating suppressive Tregs and employs a proper Treg function [85,86], it exhibits reduced efficiency in inducing or sustaining the expression of multiple genes that reinforce FOXP3 expression, leading to decreased Treg stability [92]. As exon 7 encodes part of the LZ domain, both FOXP3-ΔE7 and FOXP3-ΔE2ΔE7 lack interactions with cofactors such as FOXP1, probably impairing a critical mechanism for immunosuppression [78,93,94,95,96]. FOXP3-ΔE2 and FOXP3-ΔE7, each lacking one of the two nuclear export signals (NESs), show moderately higher nuclear localization than FOXP3-FL in transfected primary T cells. The FOXP3-ΔE2ΔE7 accumulates completely within the nucleus [97].

These isoforms exhibit distinct expression patterns in health and disease [78,79]. Peripheral blood Tregs from healthy donors predominantly express the FL variant, whereas patients with multiple sclerosis display a significant shift towards truncated variants (∆2 and/or ∆7), alongside reduced total FOXP3 expression and Treg numbers [88]. Healthy humans express all isoforms with respective ratios depending on TCR stimulation and cytokine activation. Yet individuals exclusively expressing FOXP3-ΔE2 or FOXP3-ΔE7 develop fatal autoimmune disorders [79,92,98,99,100]. Several clinical studies revealed an association between *FOXP3* splicing dysregulation and the pathogenesis of autoimmune diseases [79,101,102,103].

Alternative splicing of *FOXP3* generates FOXP3-FL and FOXP3-ΔE2 isoforms in several mammalian species, including humans [85], non-human primates [104], and domestic house cats [105], but not in rodents [78,79,84]. Emerging evidence indicates that TCR activation signals, Treg origin (thymic vs. peripheral), metabolic states, and cytokine signaling may influence the expression profiles of FOXP3 isoforms [79,85,93,106,107]. Furthermore, the complexity of the human *FOXP3* locus far exceeds that of the current annotation, evidenced by the recent discovery of a novel Treg-specific alternative promoter and its derived non-coding transcript isoform “longFOXP3”, which may fine-tune FOXP3 expression levels through transcriptional interference [108]. This evolutionary pattern suggests increased isoform complexity fine-tunes immune responses, implying FOXP3 has evolved functional adaptations that calibrate Treg suppressive activity to diverse immunological environments under selective pressure.

## 3. FOXP3 Structure and Sequence Recognition Preference: Mechanisms in Dimeric/Multimeric Ensembles, DNA Bridging and Chromatin Interactions

FOXP3, the master regulator of Treg function, primarily mediates DNA binding through its FKH domain, which is a conserved structural feature among approximately 50 FOX transcription factors [77,109,110,111]. The C-terminal FKH domain contains the nuclear localization signal (NLS) required for FOXP3 nuclear translocation [112]. Most FOX proteins’ FKH domains adopt a winged-helix conformation that recognizes the FKH consensus motif (FKHM, TGTTTAC) [82,113]. Beyond the FKH domain, FOXP3 possesses a ProR recruiting multiple cofactors, a ZF region with putative DNA-binding capacity, and an LZ domain mediating anti-parallel dimerization [95,114]. Among these domains, the FKH domain exhibits the highest mutation frequency in IPEX patients and represents the most extensively characterized region [2,115,116]. Despite extensive research on FOXP3’s structure and DNA-binding mechanism, its molecular functions, direct target genes, and in vivo specific sequence remain incompletely resolved [36,40,46]. This section combines structural insights into FOXP3 with its sequence recognition preference, summarizing observations from recent studies.

### 3.1. Domain-Swapped Dimers: An Initial Characterized Structure for DNA Binding and Long-Range Chromatin Interactions

An early identified structural feature of FOXP3 was shown as domain-swapped dimers [117,118]. Crystallographic analysis of the NFAT1: FOXP3: DNA complex demonstrated that the FOXP3 FKH domain formed a domain-swapped dimer characterized by extensive structural element exchange (helix H3, strands S2 and S3) between two FOXP3 monomers. This dimer concurrently engages two distal DNA-binding sites, bridging two separate DNA molecules in an anti-parallel orientation, a distinctive optimization unique to FOXP3 (relative to FOXP2, FOXP3 exhibits a stronger NFAT binding affinity and enhanced dimer stability) [117]. Dimerization is an intrinsic property of FOXP3 that does not require the NFAT cofactor and precedes DNA binding [117,118,119]. The dimer assembly occurs at the protein level without DNA involvement [113].

Dimerization-defective mutations at the interface (including IPEX-related mutations) substantially impair FKH domain dimerization without impairing FOXP3’s DNA-binding capacity. Such mutations differentially affect FOXP3 target gene regulation and impair its T cell suppressive function [117]. FOXP3 reorganizes chromatin interactions at loci including *PTPN22*, coordinating Treg-associated gene expression. Thus, it was initially speculated that FOXP3 mediates long-range chromatin interactions, potentially as part of its mechanism of transcriptional regulation in Tregs [118].

### 3.2. Head-to-Head Dimers: Defining the Physiological Structure and Unique Transcriptional Mechanism of FOXP3

Before 2022, FOXP proteins were widely considered to adopt the domain-swapped dimer conformation for function [113]. However, this conclusion was based exclusively on the truncated FKH domain structural data [117]. Treg-specific chromatin interactions are formed independently of FOXP3 domain-swap dimerization [120]. Furthermore, FOXP2 has been crystallized in both non-swapped monomeric and domain-swapped dimeric states, raising doubts about the physiologically relevant structure of the FKH domain [42,117,121]. The structural organization of FOXP3 outside its FKH domain, as well as the mechanistic basis for its unique functional distinction from other FOX proteins, remains unclear at that time [122].

Unlike earlier studies with the isolated FKH domain [117], in 2022, structural determination using extended sequences spanning the ZF to the FKH domain revealed a head-to-head (H-H) FOXP3 homodimer, with each monomer binding to a FKHM, respectively [122]. Subsequent analyses confirmed that this H-H dimer represents FOXP3’s physiological conformation, while the domain-swapped dimer reflects an abnormal conformation associated with pathological states [122].

Specifically, FOXP3 FKH adopts a non-swap monomeric conformation in the accompaniment of its upstream linker RUNX1-binding region (RBR), while isolated FKH without RBR forms as a swap dimer. This suggests that the RBR stabilizes the non-swap monomeric by shielding hydrophobic residues that would otherwise be solvent-exposed [122]. Following DNA binding, the RBR loop (residues 321–336) mediates H-H dimerization through hydrophobic interactions, occupying two consecutive major grooves of the DNA strand [122]. This transcriptionally active H-H dimerization confers FOXP3 with unique DNA-binding specificity, enabling its preferential recognition of inverted repeat FKH motifs (IR-FKHM), which the domain-swap dimer fails to recognize. Additionally, FOXP3 can also bind diverse suboptimal sequences by anchoring one monomer to a consensus FKHM while the other engages non-consensus sites [122]. This is consistent with a previous finding that FKH could bind specifically to two completely distinct DNA motifs [123]. Notably, H-H dimerization is exclusive to FOXP3 within the FOXP family. Owing to RBR sequence differences, FOXP1/2/4 cannot form H-H dimers and exist as monomers. This distinction may underlie the unique role of FOXP3 in Treg cell identity [122].

In contrast, truncation of RBR or disease-associated mutations (e.g., R337Q, linked to IPEX syndrome) induces FOXP3 to adopt the domain-swap dimer conformation [122]. This conformation, previously erroneously assumed to be the physiological form of FOXP3 [117], impairs DNA binding and mediates a partial loss of Treg cell functionality [122,124].

### 3.3. Multimeric Ensembles: Ultrastable Complexes for Microsatellite Recognition and DNA Bridging

In vitro structural and biochemical studies demonstrated that FOXP3 preferentially binds IR-FKHM sequences [122], raising the question of whether IR-FKHM constitutes the genuine cellular binding site for FOXP3. However, analyses of FOXP3-occupied genomic regions using chromatin immunoprecipitation followed by sequencing (ChIP-seq) and cleavage under targets and release using nuclease sequencing (CUT&RUN-seq) did not detect enrichment of IR-FKHM motifs within cells [46,56,122,125]. In addition, the canonical FKHM was detected only in a minority of FOXP3 binding sites [125]. Even among these few sites, most FKHM did not constitute binding sites for FOXP3 itself. Instead, they were bound by another family member, FOXO1, which preferentially associated with FKHM-containing enhancers in Treg precursor cells. The majority of FOXP3 binding sites were enriched for motifs recognized by its cofactors, including members of the ETS and RUNX families of transcription factors [125]. Consequently, earlier studies based solely on in vitro structural and biochemical approaches had significant limitations along with experimental artifacts.

Recent studies resolved the long-standing controversy regarding FOXP3’s in vivo binding motifs [126,127]. T*_n_*G (*n* = 2–5, particularly T_3_G) repeat microsatellites were identified as FOXP3’s primary targets, substantially surpassing the enrichment of canonical FKHM TGTTTAC [126]. De novo motif analysis of prior FOXP3 ChIP-seq and CUT&RUN-seq data further demonstrated significantly higher enrichment scores for T*_n_*G-repeat motifs relative to FKHM [46,56,120,126]. Consistent with these findings, it has been reported in a preprint that FOXP3 exhibits preferential binding to shorter TGTTT motif variants, indicating that sequence recognition preference of FOX transcription factors cannot be inferred solely from structural similarities within the FKH domain [128]. Indeed, transcription factor preference for short tandem repeats (STRs; consecutively repeated units of one to six nucleotides) is widespread [129]. Transcription factor-preferred STRs do not necessarily resemble known binding motifs [129,130].

In contrast to IR-FKHM, T*_n_*G repeat DNA induces FOXP3 multimerization. After binding to T*_n_*G repeat microsatellites, the FKH domain facilitates FOXP3 higher-order multimer formation [126]. Cryo-electron microscopy analysis of the FOXP3-T_3_G repeat complex reveals a ladder-like architecture. This structure comprises two double-stranded DNA molecules forming “side rails”, bridged by five pairs of FOXP3 molecules, each pair constituting a “rung”. Within T_3_G repeats, each FOXP3 subunit occupies TGTTTGT, mirroring its canonical DNA-binding mode (bound to the FKHM TGTTTAC) [126].

Mutations in the intra-rung interface impair T_3_G binding, DNA bridging, and FOXP3-mediated cellular functions (e.g., CTLA-4 and CD25 induction, T cell suppression), without affecting IR-FKHM binding [126], while the inter-rung interaction involves RBR-RBR contacts, consistent with H-H dimerization described in the preceding subsection [122,126]. Mutations in RBR impaired FOXP3 binding to both T_3_G repeats and IR-FKHM [126]. Cross-species and paralog analyses confirm conservation of this T*_n_*G binding mode in FOXP3 orthologs (human, platypus, zebrafish) and FOXP family members (FOXP1/2/4), suggesting it likely represents an ancient property of FOXP transcription factors [126,131].

Building on this foundation, further study elucidated FOXP3’s adaptability to T*_n_*G sequence variability through resolving the structures of FOXP3 in complex with T_2_G/T_4_G repeats [127]. T_4_G repeats induced asymmetric multimer formation, bridging 2–3 DNA molecules in distinct anti-parallel and parallel modes. In both arrangements, FOXP3 subunits exhibited identical binding to each DNA molecule, recognizing every other TGTTTTG sequence, while T_2_G repeats induced the assembly of a barrel-like complex consisting of 4 DNA molecules and 28 FOXP3 subunits, where all subunits bound every other TGTTGTT sequence [127]. This structural plasticity derives from the RBR of FOXP3, whose hydrophobic and aromatic-rich residues endow it with conformational adaptability to different T*_n_*G repeats [127]. Critically, nucleosomes facilitate FOXP3 assembly by inducing local DNA bending, which reduces the spatial distance between distal T*_n_*G repeats and establishes nucleation sites for *cis/trans* DNA bridging [127]. Such higher-order assemblies mediate long-range chromatin interactions, associating with enhancer-promoter loops of Treg-signature genes (e.g., *IL2RA*, *CD28*, and*ETS1*) [40,120,126].

Synthesis of early and recent research findings reveals that FOXP3 associates with genomic DNA as both H-H dimers and multimers in Tregs [122,127,132]. Specifically, FOXP3 recognizes multiple DNA motifs (beyond IR-FKHM) paired with FKHM via H-H dimerization. T*_n_*G repeats predominate in FOXP3 ChIP-seq peaks (~50%), contrasting with H-H motifs (~10%) [132]. The H-H sites are frequently found adjacent to T*_n_*G repeats, stabilizing FOXP3 multimerization on short, suboptimal T*_n_*G repeats through H-H dimerization [132]. While multimerization is a conserved feature of FOXP proteins [126], H-H dimerization is FOXP3-ortholog specific [122,132]. These structural features and DNA-binding modes, while initially elucidated in murine models, are conserved between murine and human FOXP3, underscoring their essential role in Treg biology [126].

Collectively, FOXP3 adopts conformationally flexible states dictated by the DNA sequence (Table 1) [122,126,127,132]. These findings extend prior evidence that FOXP3 associates with enhancer-promoter loops and is critical for establishing the Treg-specific 3D chromatin architecture [40,120]. H-H motifs complement T*_n_*G repeats to expand FOXP3’s architectural control across genomic loci, enhancing its maintenance of the Treg transcriptional program [126,127,131,132].

## 4. FOXP3-Mediated Dynamic Regulation of Treg Identity and Functional Plasticity: Coordination with Multiple Partners

FOXP3 is the key transcriptional regulator for sustaining Treg lineage identity, yet its function extends beyond autonomous DNA binding. As revealed by cumulative data over the past decade, FOXP3 does not act as an autonomous regulator by directly binding to chromatin. Instead, it exerts regulatory functions through interactions with diverse context-dependent cofactors, thereby enabling dynamic modulation of Treg identity and plasticity [45,46,125,133,134,135,136]. This chapter mainly focuses on these mechanisms as follows.

The initial discussion over how FOXP3 determined Treg lineage identity raised a key question: whether FOXP3 specifies the Treg lineage by establishing new enhancers or using pre-existing enhancer landscapes [125], as lineage-defining transcription factors often set up novel enhancer repertoires [137], whereas some activation-induced transcription factors employ pre-established enhancers [138]. Examination of chromatin accessibility at FOXP3-bound enhancers in Tregs and their precursors revealed that FOXP3 predominantly binds to pre-accessible enhancers in CD4^+^ FOXP3^−^ T cells, rather than establishing a novel enhancer landscape [125]. Analysis of DNase I hypersensitive site sequencing (DNase-seq) and ChIP-seq data showed that 98% of FOXP3-binding sites in Tregs corresponded to chromatin regions already pre-accessible in precursor CD4^+^ FOXP3^−^T cells [125]. Only 2% of these regions represented Treg-specific sites, including the CNS2 enhancer within the FOXP3 locus, which is critical for stabilizing FOXP3 expression [54]. These pre-accessible enhancers are occupied by cofactors such as ETS and RUNX family proteins, which facilitate FOXP3 recruitment and subsequent modulation of gene expression programs [125]. FKH transcription factor FOXO1 acts as a “predecessor” at FOXP3-binding sites in precursors, with its displacement in Tregs by FOXP3 downregulating proximal genes [125,139]. Furthermore, approximately 80% of FOXP3-bound sites are constitutively accessible across diverse cell types, ranging from stem cells to B or myeloid cells [140]. Most of the remaining targets gain accessibility after the double-positive thymocyte stage during T cell development before FOXP3 induction [46,140]. Thus, FOXP3, acting as an opportunistic transcriptional regulator, specifies Treg lineage by exploiting already accessible chromatin regions [125,140].

As described in the previous chapter, FOXP3 mediates long-range chromatin interactions and associates with enhancer-promoter loops [40,120,126,132]. However, FOXP3 does not directly alter chromatin accessibility [125]. FOXP3 lacks the transactivation domain, a key feature associated with chromatin opening in FOX family transcription factors [113]. It has been shown that FOXP3 indirectly regulates chromatin accessibility, employing TCF1 as one of its critical intermediaries [46]. Many FOXP3-regulated genes exhibit no direct FOXP3 binding at their promoters or enhancers [80,81]. Therefore, most of FOXP3’s interaction with DNA is indirect, mediated through protein–protein interactions [125]. It was reported that FOXP3 functions as a scaffold for DNA binding, mediated by its ProR interaction with HDACs, independently of FKH’s DNA binding. Specifically, FOXP3’s ProR recruits class I HDACs to target gene promoters (e.g., *IL2* and *IFNG*), counteracting activation-induced histone hyperacetylation and thereby repressing expression [141]. Consistent with this view, FOXP3’s interaction with RUNX1 and NFAT is essential for its function. Mutations disrupting these interactions compromise FOXP3-mediated suppression of IL-2 production and impair activation of target genes (e.g., *IL2RA, GITR,* and *CTLA4*) [42,43,142]. Additionally, FOXP1 forms heterodimer complexes with FOXP3 through interactions of the LZ domain [94,95]. FOXP1 occupies numerous FOXP3-bound genomic sites in both Treg and Tconv cells. In Tregs, FOXP1 augments FOXP3 binding to these sites and is essential for enforcing FOXP3-mediated gene regulation, demonstrating its non-redundant function [143,144]. Indeed, an increasing number of transcriptional regulators and enzymes interacting with FOXP3 have been identified, indicating that multiple partners are crucial for FOXP3’s transcriptional regulatory function [39,42,90,91,133,136,145,146,147,148,149,150,151,152].

Biochemical and mass-spectrometric analyses in 2012 identified that FOXP3 functions in large multiprotein complexes (>400 kDa) comprising 361 associated proteins, 30% of which participate in transcriptional regulation [133]. This network includes numerous sequence-specific transcriptional regulators such as GATA-3, FOXP1, NFAT, STAT3, ETS, and RUNX1 [42,43,133,136,148,150,152]. Notably, FOXP3 directly regulates about 50% of its cofactors, indicating that FOXP3 mediates transcription control through a coregulatory protein network with its partners [125,133]. Some partners (e.g., GATA-3), whose gene expression is affected by FOXP3, reciprocally promote FOXP3 expression, establishing a reciprocal regulatory circuit [133,153,154]. Specific FOXP3 partners are activated in distinct inflammatory and tissue microenvironments. Their recruitment into multimeric FOXP3 complexes integrates environmental signals, thus driving functional adaptations in Tregs, including homeostasis, homing, and effector capabilities [133]. Thus, FOXP3 defines the transcriptional network of Tregs through cooperative action with its protein complexes [155,156]. Subsequent work in 2017 further supports the view that FOXP3’s transcriptional activity, following enhancer binding, is determined by its interaction with distinct multimolecular complexes [45]. FOXP3 activates transcription when complexed with RELA/TIP60/IKZF2/EP300 (also known as p300) [147], but is inactive when bound to IKZF3/YY1/EZH2 [44,45,157,158]. Cell state, organismal location, or environmental cues may influence the balance between these two complexes, potentially via post-translational modifications (PTMs; see the next chapter for details), thereby modulating FOXP3 activity [45]. Therefore, Treg cell identity and diversity arise not solely from a monomorphic transcription factor but derive from a framework of context-dependent transcription factors. Within this framework, FOXP3 amplifies the pre-existing Treg identity, differentially regulating transcription in conjunction with distinct cofactors [37]. The difference between FOXP3 activating and repressing its target genes is determined by the cofactors it recruits at each locus [37,45]. FOXP3 exhibits context-dependent requirements across the Treg developmental stage (newly generated vs. mature Tregs) and environmental conditions (steady state vs. inflammatory stress or tumor microenvironments (TME)) [159,160,161,162]. These studies established FOXP3 functions as a multimodal interaction hub, integrating environmental signals through diverse cofactor interactions to fine-tune Treg identity and function [37,45,68,133,163,164].

For many years, FOXP3 has been widely regarded mainly as a DNA-binding transcription factor [111], and its gene regulation was thought to be static and constitutive [2,25]. Recent studies have revealed that FOXP3-chromatin interactions are not static but dynamically modulated by Treg activation status and environmental cues [134,135,165]. Proximity proteomics identified over 1400 proteins highly enriched in proximity to FOXP3, spanning diverse functions encompassing chromatin modification/remodeling, DNA topology and methylation, transcriptional regulation, and RNA splicing and exportation [134]. Of these, 157 proteins (43.5% of 361 known FOXP3 interactors) had been previously reported to interact with FOXP3 [45,133]. These findings highlight the complexity of the FOXP3-mediated regulatory network and its ability to adapt to environmental cues. CUT&RUN-seq identified three distinct FOXP3-binding modes (constitutive, increased, or decreased) in resting Treg (rTreg) and activated Treg (aTreg) cells [134]. Less than 23% of dynamic FOXP3 peaks localize to gene promoter regions, compared to 58.6% of constitutive peaks. This indicated that FOXP3 primarily engages promoters through static binding, while its association with distal regulatory elements (e.g., enhancers) preferentially undergoes dynamic regulation during Treg activation [134].

Consistent with earlier ChIP-seq data [125], significant enrichment of ETS-family binding motifs was observed in regions with constitutive FOXP3 binding. CRISPR deletion of *ETS1* in Tregs reduced FOXP3 association with chromatin, suggesting that ETS proteins facilitate constitutive FOXP3-chromatin binding to maintain basal Treg functions [134]. Acute stimulation of rTregs with IL-2 or TCR/co-receptor induced distinct FOXP3-chromatin binding profiles [134]. TCR stimulation promotes FOXP3 binding primarily at regions enriched with AP-1 motifs, a process that also functionally requires NFAT activity, whereas IL-2 stimulation facilitates binding at the STAT5 motifs [134,165]. In addition, tumor-infiltrating Treg (tuTreg) cells, which suppress anti-tumor immunity [166], exhibit distinct FOXP3-binding patterns compared to lymphoid organ-derived aTregs [134]. These observations indicate that FOXP3 “senses” environmental cues through signal-dependent transcription factors, which recruit FOXP3 to specific chromatin loci. The downstream gene expression triggered by FOXP3-chromatin interactions is determined by environmental stimuli or collaborating DNA-binding proteins [134,165]. These findings support a revised model in which FOXP3 primarily functions like a transcriptional cofactor. In this model, its chromatin binding is predominantly determined by context-specific DNA-binding proteins to dynamically regulate Treg function, while direct FOXP3-DNA interaction serves a secondary stabilizing role [135].

**Figure 1:**
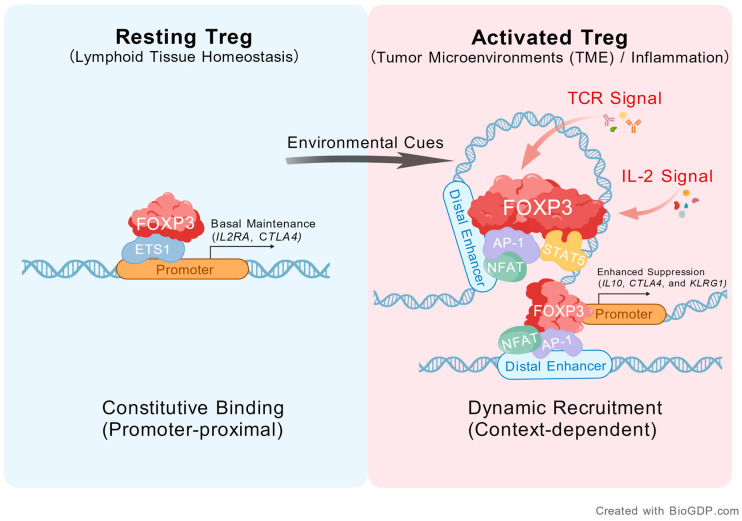
FOXP3 associates with distinct cofactors in a context-dependent manner (created with BioGDP.com [174]). FOXP3 acts on gene promoters mainly through ETS1-mediated constitutive binding to maintain basal Treg functions by regulating genes such as *IL2RA* and *CTLA4*, while its association with distal enhancers undergoes dynamic regulation during Treg activation or in TME. TCR stimulation promotes FOXP3 binding with AP-1 and NFAT, whereas IL-2 stimulation facilitates its binding at the STAT5 motifs. Under stimulation or differentiation, FOXP3 associates with chromatin via these context-dependent DNA-binding proteins to modulate gene expression (e.g., *IL10*, *CTLA4*, and *KLRG1*), which enhances the Treg suppression. Gene and protein symbols in the figure follow the human nomenclature to denote conserved mechanisms. Schematic shapes represent upstream environmental stimuli, including TCR ligands (e.g., antigens or co-stimulatory molecules) and cytokines (e.g., IL-2).

Specifically, FOXP3 associates with distinct cofactors in a context-dependent manner. In rTregs, it interacts with pre-existent ETS1 to sustain Treg basal function through regulation of genes such as *IL2RA* and *CTLA4* [125,134,167]. While in an activated or TME state, it switches to partner with AP-1 family proteins (e.g., BATF and JUNB) and NFAT to mediate immunosuppression-associated gene expression (e.g., *IL10*, *CTLA4*, and *KLRG1*) (Figure 1) [134,135,165]. BATF, which has been established as critical for Tregs differentiation and function [168,169,170,171,172,173], facilitates FOXP3-chromatin binding through its own DNA-binding capability. BATF-FOXP3 cooperative interaction moderately upregulates key suppressive molecules, including CD25 and CTLA-4 [134,165].

Although FOXP3 binds DNA with sequence specificity, it elicits transcriptional changes in a minority of the genes it occupies [46,159]. FOXP3 binding sites exhibit minimal enrichment for canonical FKHM but are predominantly characterized by the presence of cofactor (AP-1, RUNX, and ETS) motifs [125]. The FKH domain of FOXP3, previously considered to primarily mediate direct DNA binding [82,84,111,113,119], has been shown by recent studies to also play a critical role in protein–protein interaction [42,117,122,126]. FOXP3 employs a substantial proportion (64%) of its FKH domain surface area for protein–protein interactions, significantly exceeding the area used for DNA binding (19%) [127]. Direct DNA binding, when it occurs, functions primarily to stabilize FOXP3-chromatin interaction rather than determining target specificity [134].

This functional model is supported by earlier observations: even after deletion of the NLS within FOXP3’s FKH domain, the RORγt, which was induced in Tregs by food antigens or the microbiome [175], binds FOXP3, mediates its nuclear translocation, and ultimately modulates the expression of RORγt target genes [91]. Some FOXP3 mutations may impair Treg function by changing the chromatin binding of associated proteins, thereby perturbing gene expression, rather than directly compromising FOXP3-DNA interactions [45,168]. Consistent with these observations, mutations targeting the DNA-binding residues of FOXP3 diminish its affinity for FKHM and (T_3_G)_6_ motifs, but do not affect CD25 and CTLA-4 expression in overexpression systems, on the condition that the protein levels remain comparable to those of wild-type FOXP3. These findings suggest that the direct FOXP3-DNA binding may stabilize FOXP3-chromatin interaction but does not dictate core regulatory functions [134].

Collectively, these studies reveal that FOXP3 functions through a highly dynamic, context-dependent network of protein partners and chromatin interactions (mechanisms largely elucidated in murine systems) [133,134,135,136,165]. The Treg lineage-defining transcription factor FOXP3, like a transcriptional cofactor rather than a traditional direct DNA-binding transcription factor, exhibits a unique response mechanism to adapt to diverse immune demands [125,133,134,135].

## 5. Post-Translational Modifications of FOXP3: Regulation of Transcriptional Complexes Stability

As described above, FOXP3 mediates transcriptional repression or activation of target genes not by itself, but through forming multiprotein complexes [45,133,155,156]. The diverse components of these complexes modulate DNA-binding affinity and interaction modes [78]. Furthermore, FOXP3 cooperates with chromatin-modifying enzymes to epigenetically stabilize the Treg cell phenotype and function [1,30,80]. Notably, FOXP3 cofactors exhibit species- and context-specificity, partially regulated by PTMs that influence the complex formation and stability [78]. Major PTMs for FOXP3 include ubiquitination, phosphorylation, and acetylation [16,30,57,62,136].

Ubiquitination drives diverse regulatory outcomes. During infection, an effective anti-pathogen response requires a rapid suppression of Treg cell function, and the ubiquitination of FOXP3 is a key signal mediating this rapid Treg inhibition [30,176]. The E3 ubiquitin ligase STUB1 induced by inflammatory stimuli interacts with FOXP3. Together with the chaperone HSP70, STUB1 catalyzes K48-linked polyubiquitination of FOXP3, targeting its proteasome degradation and consequently impairing Treg suppressive function [136,176]. Distinct from STUB1, TRAF6-mediated K63-linked polyubiquitination facilitates the proper nuclear localization of FOXP3 and enhances its transcriptional activity in Tregs [177]. RNF31 catalyzes the atypical ubiquitination of FOXP3, promoting its protein stability and reinforcing Treg suppressive function [178,179]. USP7, USP21, USP22, and USP44 interact directly with FOXP3 to prevent its degradation by deubiquitinating the K48-linked polyubiquitin chains [180,181,182,183,184,185]. Among them, USP7 enhances the FOXP3-TIP60 interaction, thereby maintaining FOXP3 expression [180,181]. USP21 inhibits Th1-like phenotypes while sustaining expression of Treg signature genes [182]. It also interacts with GATA3, potentially forming a positive regulatory loop with GATA3 and FOXP3 to promote FOXP3 expression and modulate Treg activity [183]. USP44 stabilizes FOXP3 in cooperation with USP7 during the deubiquitination process [185].

The acetylation of specific FOXP3 lysine residues, mainly catalyzed by EP300/CREBBP (also known as p300/CBP) and TIP60, enhances FOXP3 stability and its DNA-binding ability [186,187], while activating specific effector functions [188]. TIP60 and EP300 cooperatively regulate FOXP3 activity [62,189,190,191]. This process competitively inhibits ubiquitination at identical residues, thereby blocking the ubiquitin-dependent proteasomal degradation of FOXP3 [187]. TIP60, EP300, and HDAC7 form a functional complex with FOXP3, promoting its acetylation and targeting the N-terminal ProR, which is crucial for IL-2 repression [39,187]. Meanwhile, through the addition and removal of acetyl groups on histones, the FOXP3 complex realizes chromatin accessibility modifications to other transcription factors [30,39]. HDAC3, HDAC6, HDAC9, and SIRT1 deacetylate FOXP3, regulating its function by modulating the interactions with different transcription factors [39,57,62,192,193]. These deacetylases have both shared and different mechanisms, and the combined loss of their activity can enhance Treg function [193,194,195].

Phosphorylation has bidirectional regulatory effects on FOXP3 function. Kinase NLK positively modulates FOXP3 activity, whereas CDK2, PIM1, and PIM2 negatively regulate its function [62]. NLK enhances FOXP3 stability by preventing its ubiquitin-dependent degradation [57]. TCR stimulation can activate the TAK1-NLK signaling [76], leading to the phosphorylation of FOXP3 at multiple residues, which reduces its interaction with STUB1 and regulates the degradation rate of FOXP3 [196]. CDK2-mediated phosphorylation at Ser19/Thr175 leads to diminished FOXP3 protein stability and Treg suppressive function [197,198]. PIM1 does not affect FOXP3 stability but impairs its DNA-binding activity [57]. Under inflammatory conditions, PIM1 phosphorylates FOXP3 at Ser422 within the FKHM, attenuating DNA binding and Treg function [199]. PIM2 does not compromise protein stability either, but disrupts the interaction between FOXP3 and other cofactors [57]. It phosphorylates FOXP3 at multiple sites within the N-terminal ProR, which is crucial for binding partners including IKZF4 (also known as EOS), TIP60, and HDAC7 [57,62,200].

The activity of FOXP3 protein can also be regulated by other PTMs, including glycosylation and methylation [57,62]. Glycosylation of FOXP3 mainly involves TCR-activated O-linked N-acetylglucosamine (O-GlcNAc) modification at serine/threonine residues, with O-GlcNAc transferase catalyzing O-GlcNAc addition and O-GlcNAcase mediating removal [201]. This reversible modification stabilizes FOXP3 by counteracting ubiquitin-mediated degradation, activates STAT5, and sustains Treg lineage stability and suppressive function [201,202]. Methylation of FOXP3 primarily occurs at arginine residues, catalyzed by protein arginine methyltransferases (PRMTs) including PRMT1 and PRMT5. PRMT1 induces asymmetric dimethylation, while PRMT5 mediates symmetric dimethylation [203,204]. These modifications are essential for maintaining Treg suppressive function.

Although the currently published data are not sufficient to fully clarify the roles of FOXP3 PTMs in regulating Treg suppressive function [62], existing evidence shows that PTMs can modulate FOXP3 protein expression levels, subcellular localization, and the stability of its transcriptional complexes (mechanisms largely elucidated in murine systems) [30,57,136]. These PTMS can respond dynamically to microenvironmental signals, which may affect the fate of each FOXP3^+^ cell [62,135,136].

## 6. Discussion

Following over two decades of intensive research, it is now recognized that the Treg lineage-defining transcription factor FOXP3 exhibits unique characteristics distinct from other T cell transcription factors and, in fact, most transcription factors overall.

(1) Unlike conventional lineage-defining regulators, FOXP3 itself does not substantially alter chromatin accessibility nor establish new enhancers. Instead, it defines Treg functionality by primarily exploiting the pre-existing epigenetic landscape established in precursor cells during differentiation [125,140].
(2) FOXP3 exhibits unique DNA sequence recognition preferences and can form diverse multimeric structures. Unlike other FOX family members, which typically bind to the canonical FKHM [113], FOXP3 can adopt multiple distinct conformations to accommodate variable T*_n_*G sequences or H-H motifs, subsequently forming stable multimers that facilitate DNA bridging and the stabilization of chromatin loops [126,127,132]. Although not a pioneer transcription factor, FOXP3 can utilize nucleosomes to enhance its DNA target recognition. Specifically, nucleosome-mediated local DNA binding between two T*_n_*G repeats promotes FOXP3 multimer assembly. This represents a previously unrecognized interaction pattern between nucleosomes and non-pioneer transcription factors [127].
(3) Many genes previously considered to be regulated by FOXP3 are not its direct targets. In fact, FOXP3 does not have clearly defined direct target genes. Treg-specific transcriptomes are only partially dependent on FOXP3. FOXP3 is neither required nor adequate to determine Treg identity [12,13,32,33,34,35,36,37]. Downstream gene expression arising from FOXP3-chromatin interactions is context-dependent, dictated by environmental cues and the specific cooperative transcription factor interactions [45,134,165]. Consequently, FOXP3 functions primarily as a multimodal interaction hub (rather than a conventional direct-acting transcription factor), integrating environmental signals through interactions with diverse cofactors to dynamically regulate gene expression [37,45,68,133,134,135,163,164,165].

The intricate functional programs orchestrated by FOXP3 keep revealing its surprising adaptability across distinct immune environments, explaining the paradoxes and unexpected observations reported over the years. For instance, ChIP-seq analyses from multiple studies demonstrated that FOXP3 associates with a large number of genomic sites distal to Treg-specific loci [45,46,56,80,81,125]. This is attributed to FOXP3’s capacity to mediate long-range chromatin interactions via its distinctive multimerization and DNA-bridging activities [40,126,127,132]. Long-range chromatin interactions refer to the physical contacts between distant genomic regions (intra- or interchromosome), forming the 3D nuclear architecture [205,206]. These interactions facilitate coordinated gene regulation by bringing regulatory elements (such as enhancers) into proximity with their target promoters, even over large linear genomic distances [205,206,207]. Enhancers and promoters can both be bound by multiprotein complexes comprising DNA-binding transcription factors and enzymatic cofactors (e.g., histone modifiers) [205]. Multiple studies have established that such long-range chromatin interactions involve protein–protein interactions and specific bridging complexes [205,208,209,210,211,212]. Cell-specific chromatin interactions establish the structural frameworks for cell-specific transcriptional programs [207]. In Tregs, which must dynamically adapt to diverse immune environments and maintain precise regulatory functions, FOXP3 acts as such an integrative bridging hub of chromatin interactions and protein complexes. Beyond stabilizing genome organization, FOXP3 integrates environmental signals through these interactions to globally fine-tune gene expression. Since FOXP3 mainly occupies pre-accessible chromatin regions, individual chromatin interaction events are unlikely to induce substantial transcriptional alterations. Instead, its binding and bridging across thousands of genomic loci may exert subtle yet global modulation of gene expression dynamics [132], consistent with its modest effects (<2–3 fold) across hundreds of genes [160].

For decades, the transcriptional role of FOXP3 (whether as an activator or repressor) has been debated. Accumulating evidence now confirms that it functions highly dependent on both context and interacting cofactors [37,45,134]. Accordingly, FOXP3 exhibits remarkable adaptability and dynamic regulatory capacity across various environmental conditions (e.g., steady state vs. inflammatory stress or TME) and cell differentiation stages (e.g., newly generated vs. mature Tregs) [134,135,159,160,161]. The TME and lymphoid organs regulate dynamic FOXP3-chromatin interactions through distinct mediators, with TME-specific regulation potentially enhancing Treg function and tumor immune evasion [134,166]. Inflammatory signals regulate FOXP3 complex stability through inducing PTMs and multiple mechanisms, thereby modulating immune response intensity and duration to prevent tissue damage [30,176,199,213,214,215,216]. These features of FOXP3 emphasize the profound regulatory multifunctionality contained in the term “regulatory” designation for Tregs.

Future research should employ advanced methods (such as rapid in vivo protein degradation systems and multiple high-throughput genomics) to analyze the complex interactions of FOXP3 with chromatin, diverse cofactors, and environmental signals. We expect these approaches to help us comprehensively understand the dynamics of FOXP3’s chromatin interactions and the context-specific FOXP3 complex networks in vivo, thereby refining the functional characterization of FOXP3 in Tregs.

## 7. Conclusions

As the lineage-defining transcription factor for Tregs, FOXP3 functions beyond its conventional DNA-binding role. Its unique sequence recognition preferences and multimeric structure mediate long-range chromatin interactions. The transcriptional outcomes are determined by the context-specific cofactors it interacts with, while the direct FOXP3-DNA interaction serves as a secondary stabilizing role. FOXP3’s ability to act as the master transcription factor of Tregs relies on its capacity to organize chromatin and recruit complexes, rather than these being merely supportive functions. Collectively, FOXP3 acts as an integrative hub, coordinating chromatin interactions and transcriptional complex formation to integrate environmental cues and dynamically orchestrate global gene expression in Tregs.

## Figures and Tables

**Table 1:** Comparative structural and functional features of FOXP3 dimeric and multimeric complexes.

Structure Type	Domain-Swapped Dimers	Head-to-Head (H-H) Dimers	Multimeric Ensembles		
Structural Features	2 subunits; domain-swapped (helix H3, strands S2/S3 exchange)	2 subunits; occupies two consecutive DNA major grooves	28 subunits in T_2_G complex; barrel-like	5 FOXP3 pairs in T_3_G complex; ladder-like	Asymmetric multimers in T_4_G complex
DNA-Binding Mode	Bridges two distal DNA molecules in anti-parallel orientation	Binds inverted repeat FKH motifs (IR-FKHM); can engage consensus/non-consensus sites	Bridges 4 DNA molecules, binds every other TGTTGTT	Anti-parallel; bridges 2 DNA molecules, binds TGTTTGT	Anti-parallel/parallel; bridges 2–3 DNA molecules, binds every other TGTTTTG
DNA Required for Assembly?	No (dimerizes at protein level independently of DNA)	Yes (dimerizes post DNA binding)	Yes (induced by T*_n_*G-repeat DNA)		
RUNX1-binding Region (RBR) Mediated?	No	Yes	Yes		
FOXP3-Specific?	No (observed in FOXP2 as well)	Yes (FOXP1/2/4 cannot form due to RBR sequence differences)	No (conserved in FOXP family)		
Function/ Pathophysiological Role	Pathological conformation (linked to autoimmune diseases)	Physiological conformation; stabilizes multimerization on suboptimal T*_n_*G repeats	Physiological conformation; Mediates long-distance chromatin contacts (enhancer-promoter loops); forms Treg-specific 3D chromatin architecture		
Data Source	[117,118]	[122,132]	[126,127]		

## Data Availability

Data available on request from the authors.

## Abbreviations

The following abbreviations are used in this manuscript:

Tregs	Regulatory T cells
IPEX	Immune dysregulation, polyendocrinopathy, enteropathy, X-linked
Tconv	Conventional CD4^+^ T
APC	Antigen-presenting cell
IL-2R	IL-2 receptor
Th	T helper
FKH	Forkhead
CREs	*Cis*-regulatory elements
CNS	Conserved non-coding sequences
TCR	T cell receptor
TGF-βR	TGF-β receptor
TSDR	Treg-specific demethylated region
SCFAs	Short-chain fatty acids
HDAC	Histone deacetylase
NS-	Negative non-coding sequence
NS+	Positive non-coding sequence
FOX	Forkhead box
ProR	Proline-rich region
ZF	Zinc finger
LZ	Leucine zipper
FOXP3-FL	Full-length FOXP3
FOXP3-ΔE2	Exon 2-deficient FOXP3 variant
FOXP3-ΔE7	Exon 7-deficient FOXP3 variant
FOXP3-ΔE2ΔE7	FOXP3 variant lacking both Exon 2 and Exon 7
NES	Nuclear export signal
NLS	Nuclear localization signal
FKHM	FKH consensus motif
H-H	Head-to-head
RBR	RUNX1-binding region
IR-FKHM	Inverted repeat FKH motifs
ChIP-seq	Chromatin immunoprecipitation followed by sequencing
CUT&RUN-seq	Cleavage under targets and release using nuclease sequencing
STRs	Short tandem repeats
DNase-seq	DNase I hypersensitive site sequencing
PTMs	Post-translational modifications
TME	Tumor microenvironments
rTreg	Resting Treg
aTreg	Activated Treg
tuTreg	Tumor-infiltrating Treg
O-GlcNAc	O-linked N-acetylglucosamine
PRMTs	Protein arginine methyltransferases
